# Elements including metals in the atomizer and aerosol of disposable electronic cigarettes and electronic hookahs

**DOI:** 10.1371/journal.pone.0175430

**Published:** 2017-04-17

**Authors:** Monique Williams, Krassimir Bozhilov, Sanjay Ghai, Prue Talbot

**Affiliations:** 1 Department of Cell Biology and Neuroscience, University of California Riverside, Riverside, California, United States America; 2 Central Facility for Advanced Microscopy and Microanalysis, University of California Riverside, Riverside, California, United States America; University of California, Merced, UNITED STATES

## Abstract

**Objective:**

Our purpose was to quantify 36 inorganic chemical elements in aerosols from disposable electronic cigarettes (ECs) and electronic hookahs (EHs), examine the effect of puffing topography on elements in aerosols, and identify the source of the elements.

**Methods:**

Thirty-six inorganic chemical elements and their concentrations in EC/EH aerosols were determined using inductively coupled plasma optical emission spectroscopy, and their source was identified by analyzing disassembled atomizers using scanning electron microscopy and energy dispersive X-ray spectroscopy.

**Results:**

Of 36 elements screened, 35 were detected in EC/EH aerosols, while only 15 were detected in conventional tobacco smoke. Some elements/metals were present in significantly higher concentrations in EC/EH aerosol than in cigarette smoke. Concentrations of particular elements/metals within EC/EH brands were sometimes variable. Aerosols generated at low and high air-flow rates produced the same pattern of elements, although the total element concentration decreased at the higher air flow rate. The relative amount of elements in the first and last 60 puffs was generally different. Silicon was the dominant element in aerosols from all EC/EH brands and in cigarette smoke. The elements appeared to come from the filament (nickel, chromium), thick wire (copper coated with silver), brass clamp (copper, zinc), solder joints (tin, lead), and wick and sheath (silicon, oxygen, calcium, magnesium, aluminum). Lead was identified in the solder and aerosol of two brands of EHs (up to 0.165 μg/10 puffs).

**Conclusion:**

These data show that EC/EH aerosols contain a mixture of elements, including heavy metals, with concentrations often significantly higher than in conventional cigarette smoke. While the health effects of inhaling mixtures of heated metals is currently not known, these data will be valuable in future risk assessments involving EC/EH elements/metals.

## Introduction

Disposable electronic cigarettes (ECs) and electronic hookahs (EHs) are popular new tobacco products [1]. Disposable ECs and EHs combine the battery and cartomizer into a single unit, which cannot be recharged [1]. Disposable ECs/EHs, which are similar in design but differ in their nicotine concentrations, flavors, and coloring, are sold in convenience stores, drug stores, gas stations, and on the Internet. Aerosols are generated from disposable ECs/EHs when the atomizing unit heats the fluid located in the chamber adjacent to the battery. EC/EH aerosols can be produced by either air-flow activation or by button-activation. Generally, ECs/EHs run out of battery power after several hundred puffs, at which time they no longer produce aerosol and are discarded.

ECs are comprised of metal components, and metals from these components are present in the aerosol of popular brands of cartomizer style ECs, raising concerns about their safety [2–4]. High concentrations of tin were detected in the fluid of a popular brand of EC, and the concentrations of some metals in one brand of EC were higher in EC aerosol than in cigarette smoke [2]. Metals found in EC fluid and aerosol have included nickel, lead, chromium, copper, zinc, and silver [2–6]. Inhalation of metals can produce unwanted health effects such as coughing, wheezing, chest tightness, shortness of breath, and metallic taste in the mouth [2,7–9]. Some of the metals detected in EC aerosols, such as nickel, and lead, are considered carcinogens that could cause more severe health effects with long term use [10].

The purpose of this study was to identify the chemical elements, including metals, and quantify their concentrations in the aerosols from popular brands of disposable ECs/EHs, examine the effect of puffing topography on elemental content in EC/EH aerosols, and determine the source of elements/metals found in EC/EH aerosols.

## Materials and methods

### Selection of disposable ECs/EHs and conventional cigarettes

Disposable ECs were purchased from local retailers, drug stores, and on the Internet. The following ECs were evaluated: Vype (British American Tobacco Company, England), BluCig (Lorillard Inc., Greensboro, NC), NJOY King (NJOY, Scottsdale, AZ), Square 82 (PHD Marketing, Inc., Pomona, CA), Mistic (Mistic ECigs, Charlotte, NC), and V2 Cig (VMR Products LLC., Miami, FL). Disposable EHs were purchased from local smoke shops and from Internet vendors. The following brands of EHs were used: Starbuzz (PHD Marketing, Inc., Pomona, CA) (this device is labeled as an EC, but Starbuzz is a hookah specific brand), Imperial Hookah (Imperial Smoke, Santee, CA), Luxury Lites (Luxury Lites, Waco, TX), Smooth (Smooth Cigs, Spring, TX), and Tsunami (Tsunami Electronic Cigarette, Troy, MI) (Table 1). To compare EC/EH aerosol to conventional cigarettes, Marlboro Red cigarettes (Altria, Richmond, VA) were purchased from local retailers. All products were stored at room temperature. At least five copies of each EC/EH model was purchased at one time to ensure that direct comparisons could be made between models within a brand.

**Table 1:** List of disposable ECs/EHs with corresponding air-flow rates used.

Brand	Battery Activation	Flavor Nicotine Conc (mg)	Low AFR First 60 (mL/s)Puff Volume (mL)	High AFR First 60 (mL/s)Puff Volume (mL)	AFR Over Time First 60 (mL/s)Puff Volume (mL)	AFR Over Time Last 60 (mL/s)Puff Volume (mL)
BluCig	Air-Flow	Classic Tobacco (24)	10 (43)	18 (77.4)	10 (43)	13 (55.9)
Mistic	Air-Flow	Menthol (24)	19 (81.7)			
NJOY King	Air-Flow	Traditional (45)	11 (47.3)	21 (90.3)		
Square 82	Button	Original Red (18)	3 (12.9)		4 (17.2)	4 (17.2)
V2 Cigs	Air-Flow	Red (18)	13 (55.9)		13 (55.9)	13 (55.9)
Vype	Air-Flow	Classic Regular (12.5)	10 (43)			
Imperial Hookah	Button	Minty Grape (0)	3 (12.9)			
Luxury Lites	Button	Citrus Berry (6)	3 (12.9)		5 (21.5)	5 (21.5)
Smooth	Air-Flow	Watermelon Punch (0)	19 (81.7)			
Starbuzz	Button	Blueberry Mist (12)	3 (12.9)			
Tsunami	Air-Flow	Cool Mist (12)	16 (68.8)			
Marlboro Red -ISO	N/A	N/A	17 (37.4)			
Marlboro Red -CS	N/A	N/A	24 (52.8)			

### Dissection of disposable ECs/EHs

Disposable EC/EH units were cut at the level of the mouthpiece to reveal the intact atomizing unit [2,3]. The Poly-fil fibers were removed using forceps, exposing the sheath and wires. For each disposable unit, the following were recorded: type of activation, flavor, nicotine concentration (Table 1), the lab inventory letter code assigned to each unit, whether the Poly-fil was centrifuged after dissection, the amount of fluid recovered upon centrifugation, fluid color, integrity of the wire, condition of the solder and wick, and evidence of use before purchase. Cartomizer dissections were photographed using a Canon SLR digital camera, and individual components were imaged using the Nikon SMZ 745 stereomicroscope.

### Aerosol preparation and analysis in disposable ECs/EHs

All EC/EH aerosols and mainstream cigarette smoke were generated using a smoking machine described in detail previously [2,11–13]. EC/EH aerosol and conventional cigarette smoke were puffed into a 500 mL round bottom flask submerged in an ice bath and the opening of the flask was covered with Parafilm to prevent escape of the aerosol. A small glass capillary tube served as an exhaust. For each brand, aerosol solutions were prepared from three fresh disposable units. For each unit, 4.3-second puffs [14] were taken every 5 minutes, and aerosol was allowed to fully dissolve in a solution of 10% nitric acid, 3% hydrochloric acid, and 87% deionized water before the next puff was added to the flask. Mainstream smoke from conventional cigarettes (Marlboro Red) was prepared as described above using the International Organization for Standardization protocol (ISO, 2.2-second puff, puff volume of 35 ml, every minute) and the Canadian Standard protocol (CS, 2.2-second puff duration, puff volume of 55 ml, every 30 seconds) [15]. The cigarettes used for the ISO and CS were purchased at different times. Room air was prepared in a similar fashion except no EC/EH was used for three samples. All aerosol/smoke/air samples were stored in 15 mL conical vials. An Optima 7300 PV (Perkin-Elmer, Waltham, MA) inductively coupled plasma optical emission spectrometer (ICP-OES) was used to quantify the concentrations of elements in each aerosol/smoke and the room air sample [3]. Details on the operation of the ICP-OES, quality control, operating parameters, and the limits of detection for each element are given in the supplementary data (S1 Material, S1 Table) and have been published previously [3]. The concentration of elements in room air samples were subtracted from the aerosol samples to determine the actual concentration in aerosol samples. Concentrations are presented as μg/10 puffs to allow comparison to the conventional cigarette smoke samples, which would be about 10 puffs.

### Effect of puffing topography on elements in EC/EH aerosols

To determine how elements change with larger puffs, aerosols were generated using higher air-flow rates. To determine how element concentrations in aerosols vary with puff number, aerosols were generated as described in the above sections from three unused units. The first 60 puffs were collected, then the devices were puffed for a particular number of puffs without collecting aerosol, and then the last 60 puffs of aerosol were collected. The number of puffs between the collection of the first 60 and last 60 puffs was determined from data in our prior study [1]. All samples of aerosol were then analyzed using ICP-OES [3].

### Elemental analysis of components in the atomizing units of disposable ECs/EHs

For each brand, dissected disposable EC/EH wires, wicks, sheaths, and the joints between the wires and batteries were mounted on aluminum pin stubs covered with carbon tape [2,3]. The morphology and elemental composition of each sample was analyzed using an FEI Co. NovaNano SEM 450 equipped with Oxford Instruments Inc. Aztec Synergy energy dispersive X-ray spectrometer (EDS) fitted with an X-Max50 50 mm^2^ SDD detector with energy resolution of 126 eV at MnK-alpha in the Central Facility for Advanced Microscopy and Microanalysis at the University of California at Riverside. Scanning electron microscope (SEM) images of samples not coated with conductive film were acquired in the secondary electron mode with a dedicated detector at 15 kV accelerating voltage. The spatial distribution of chemical elements was determined by generating elemental EDS maps using Aztec software [3]. The presence of minor elements below 1% weight in the analyzed components was determined by acquiring EDS spectra from selected points and quantifying the elemental concentrations.

### Statistical analysis of data

The mean concentration of each element in each EC/EH brand was compared to the concentration in Marlboro Red mainstream smoke prepared with the ISO protocol using a two-tailed t-test (GraphPad Prism). When Marlboro Red (ISO) had no detectable concentration of a particular element, a one group t-test was run with the value of the Marlboro Red group being set to zero. Means were considered to be significantly different when p < 0.05. When an element was below the limit of quantification, it was treated as a zero in the statistical analysis.

## Results

### Representative dissections of air-flow activated and button-activated disposable ECs/EHs

**Fig 1:**
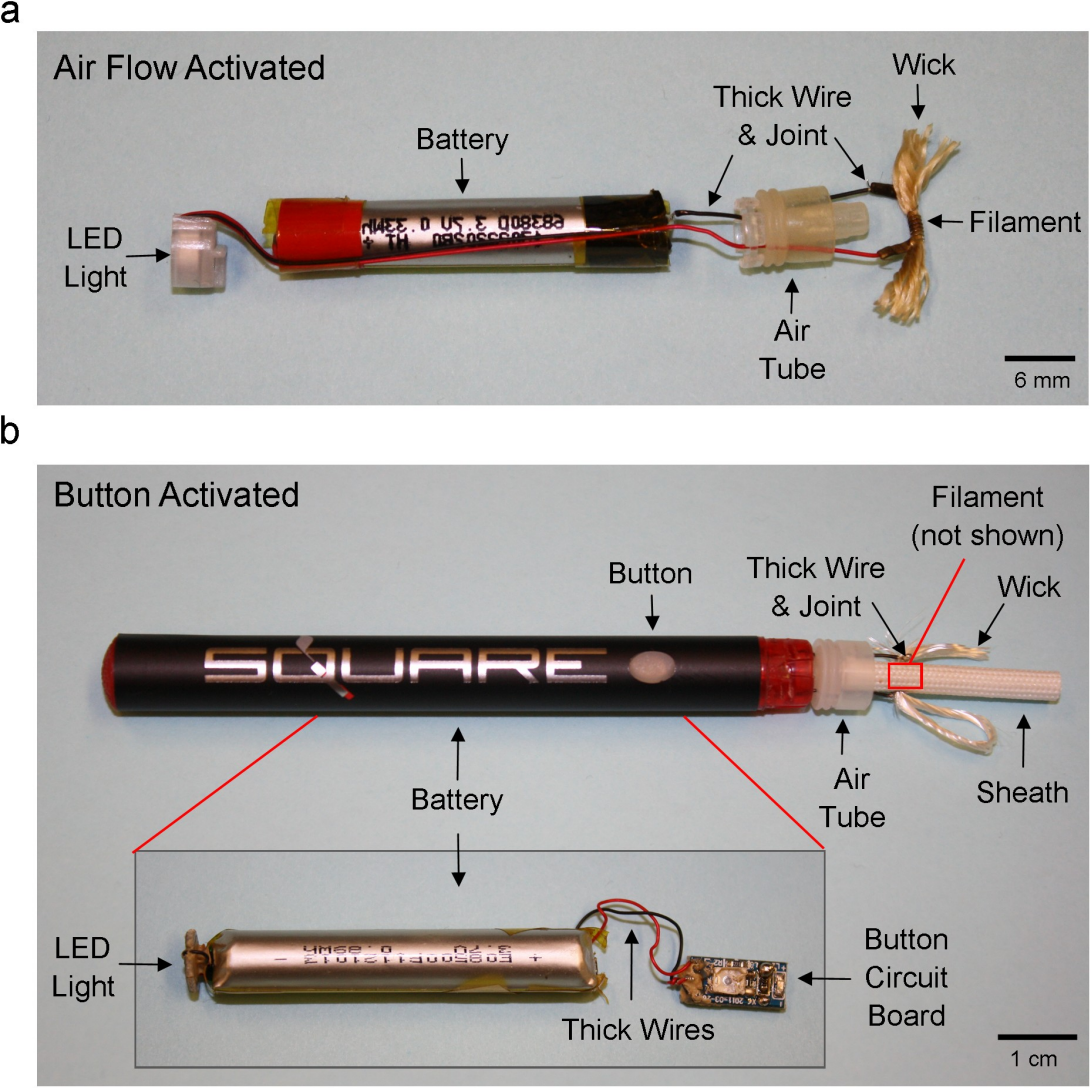
**Internal anatomy of air-flow activated (A) and button-activated (B) disposable ECs/EHs.** Both models included the LED light, battery, air-tube, thick wires, joints, wick, filament (thin wire), and sheath. The button-activated models also included the button and button circuit board. The internal anatomy of both ECs and EHs are similar.

Six disposable ECs (Vype, Square 82, NJOY King, Mistic, BluCig, and V2 Cigs) and five disposable EHs (Starbuzz, Tsunami, Imperial Hookah, Luxury Lites, and Smooth) were dissected to analyze the internal anatomy of the atomizing unit. Fig 1 shows a representative layout of the internal components of disposable air-flow activated (Fig 1A) and button-activated (Fig 1B) EC models. For air-flow and button-activated ECs/EHs, the internal components were very similar in design, and only the external shell varied in appearance. All brands had the same basic components: LED light, battery, air-tube, thick wires that joined to a thin wire (filament), wick, and one or two sheaths (not shown for air-flow activated) (Fig 1). A majority of the brands used in this study were air-flow activated models. For these brands, the air-tube was sometimes a solid piece of plastic (not shown), in contrast to the clear plastic style air-tube shown in Fig 1. The major difference between air-flow activated and button-activated ECs/EHs was the presence of an external button and underlying circuit board for activating aerosol production (insert in Fig 1B).

### Abundance of each element in individual brands of disposable EC aerosol

**Fig 2:**
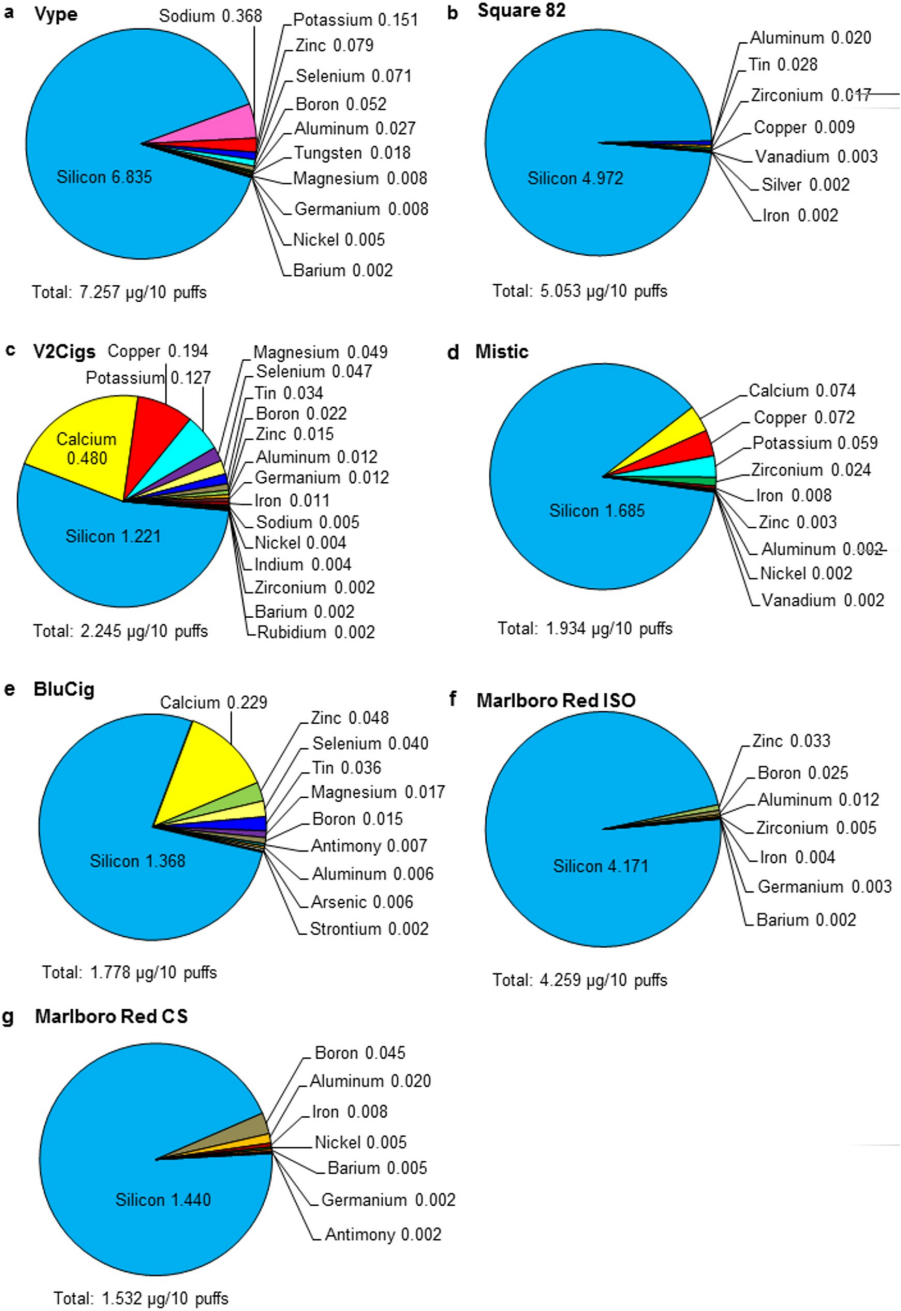
Concentration of elements in disposable EC aerosol (first 60 puffs) and in Marlboro Red cigarette smoke. The concentration of elements in aerosols from (A) Vype, (B) Square 82, (C) V2 Cigs, (D) Mistic, (E) BluCig, and in smoke from (F) Marlboro Red ISO, and (G) Marlboro Red CS are shown in the pie charts as a percentage of the total concentration of elements for each brand. The total concentration of all elements is given for each brand in μg/10 puffs beneath each pie chart. Numbers adjacent to each element are concentrations in μg/10 puffs for that element. For each brand, all concentrations are the average of three aerosol samples from three different ECs, and only elements that were higher than or equal to 0.002 μg/10 puffs are presented in this figure.

The first 60 puffs of aerosol were generated using five brands of disposable ECs (Vype, Square 82, V2 Cigs, Mistic, and BluCig). For each brand, the first 60 puffs were collected using the lowest air-flow rate that produced a robust puff (Table 1), and the concentrations of elements/metals in each aerosol were analyzed using ICP-OES [2,3]. The relative abundances of elements with concentrations above 0.002 μg/10 puffs in EC aerosols are shown in pie charts (Fig 2).

While disposable ECs are similar in design, the concentration of elements/metals in their aerosols varied within and between brands (Fig 2). The total concentration of elements/metals in EC aerosol ranged from 1.778 (BluCig) to 7.257 (Vype) μg/10 puffs (Fig 2A–2E). Silicon was the dominant element in the aerosols across all brands (Fig 2A–2E). Other elements/metals that appeared frequently in the EC aerosols at concentrations greater than 0.01 μg/10 puffs included calcium, copper, tin, potassium, boron and zinc (Fig 2A–2E), while additional elements/metals were found in trace amounts.

### Elemental abundance in disposable ec aerosol at low and high air-flow rates

**Fig 3:**
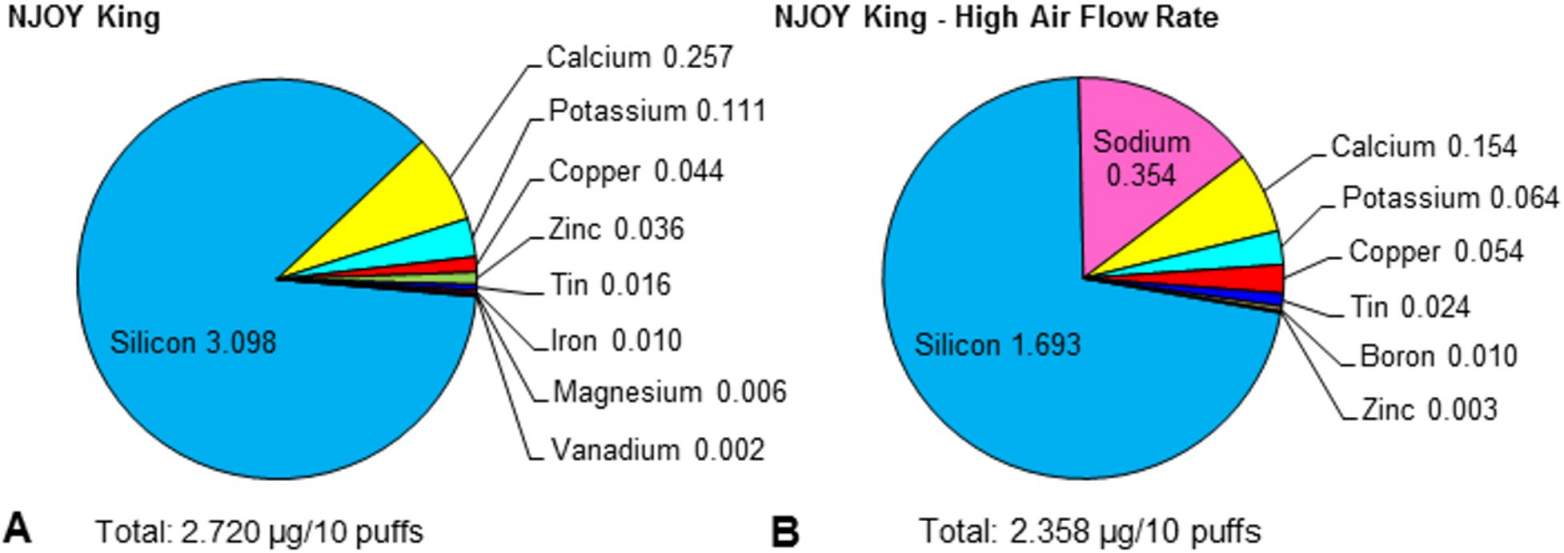
Elemental analysis of disposable ECs at low and high air-flow rates. The concentrations of elements in the aerosol of NJOY King were measured at low (A) and high (B) air-flow rates, and are shown for each element in the pie charts as the percentage of the total concentration of all elements. Sodium was not measured in the aerosol from NJOY King puffed at a low air-flow rate. The total concentration of all elements is given at the bottom of each pie chart. Numbers adjacent to each element are concentrations in μg/10 puffs for that element. All concentrations are the average of three aerosol samples from three different ECs, and only elements that were higher than or equal to 0.002 μg/10 puffs are presented in this figure.

To determine how the concentration of elements/metals in aerosols changes with air-flow rate, aerosols were generated for NJOY King using three unused units puffed at low air-flow rate (15 mL/s) and three different unused units puffed at a higher air-flow rate (21 mL/s) (Fig 3, Table 1). Increasing the air-flow rate resulted in a decrease in the total concentration of elements/metals from 3.584 to 2.358 μg/10 puffs. Disregarding sodium, which was not analyzed in the low air-flow rate samples, similar profiles were found in the samples of both the low and high air-flow rate (Fig 3). Silicon was the dominant element in the aerosol and contributed most to the overall total concentration, as was seen in the first 60 puffs for those ECs shown in Fig 2. In addition to silicon, the dominant elements/metals in this experiment were calcium, sodium, copper, zinc, tin, boron and iron. These data suggest that the air-flow rate used in this experiment did have an effect on the total concentration, but not necessarily the type of elements/metals in the EC aerosols.

### Abundance of each element in individual brands of disposable EH aerosol

**Fig 4:**
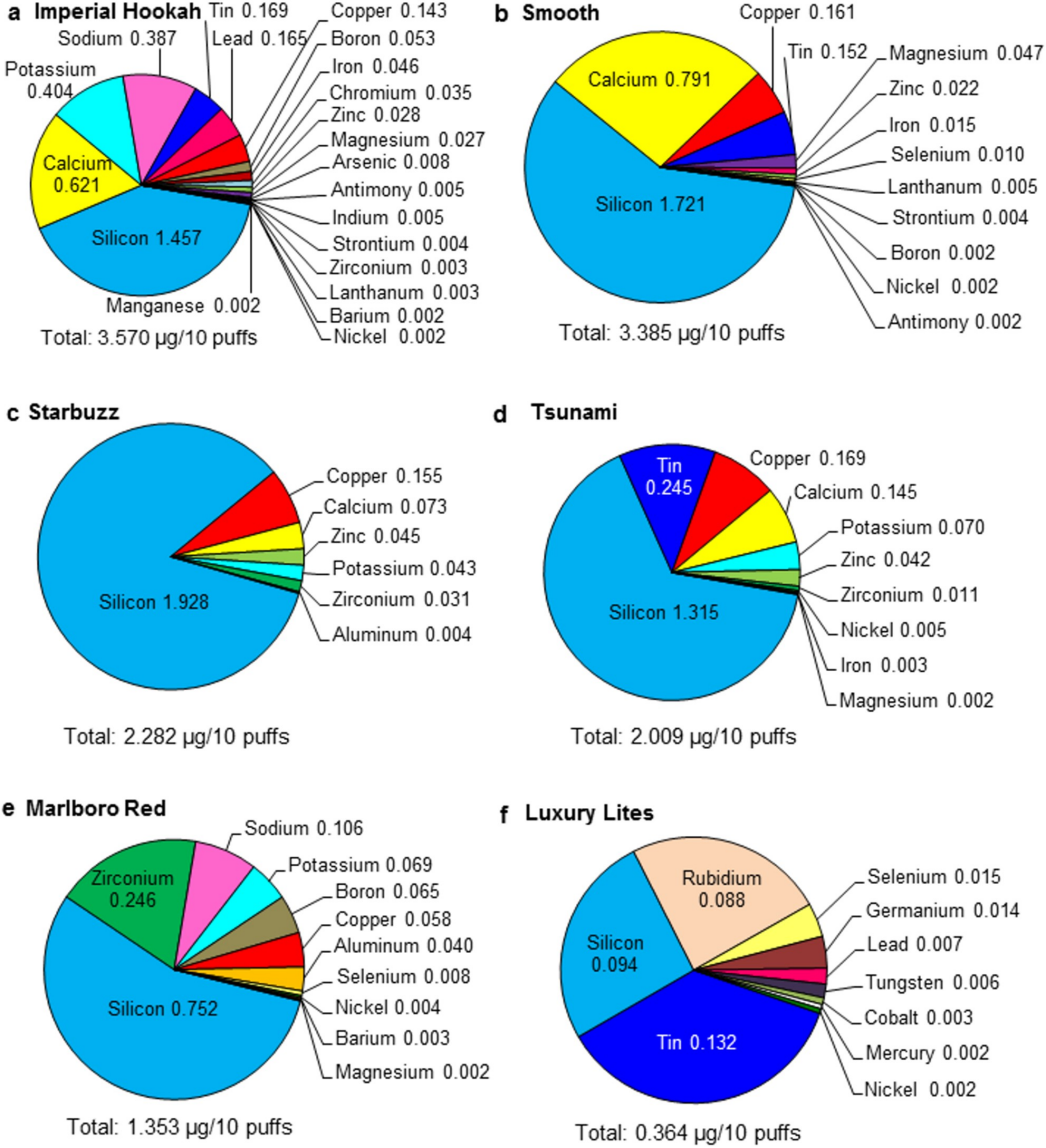
Concentration of elements in disposable EH aerosol and Marlboro Red cigarette smoke. The concentration of elements in the aerosols of (A) Imperial Hookah, (B) Smooth, (C) Starbuzz (D) Tsunami, and in smoke from (E) Marlboro Red ISO and (F) Marlboro Red CS are presented in each pie chart as a percentage of total element/metal concentration. The total concentration of all elements is given in μg/10 puffs at the bottom of each figure for each brand. Numbers adjacent to each element are concentrations in μg/10 puffs for that element. All concentrations presented are the average of three samples, and only elements higher than or equal to 0.002 μg/10 puffs are presented in this figure.

The first 60 puffs of aerosol were generated using four brands of disposable EHs (Imperial Hookah, Smooth, Starbuzz, and Tsunami). For each brand, the first 60 puffs were collected using the lowest air-flow rate that produced a robust puff (Table 1). Aerosols were analyzed as described previously using ICP-OES to determine the concentration of elements/metals present [2,3]. The concentrations of elements/metals and their relative abundance in EH aerosol are shown in the pie charts in Fig 4 for elements with concentrations above 0.002 μg/10 puffs.

The total concentration of elements/metals in EH aerosol ranged from 2.009 (Tsunami) to 3.570 (Imperial Hookah) μg/10 puffs (Fig 4A–4D). For each EH brand, silicon was the dominant element in the aerosol and contributed most to the total concentration of elements (Fig 4A–4D). Other elements/metals that were relatively abundant in EH aerosols included calcium, tin, copper, potassium, zinc, zirconium, and magnesium (Fig 4A–4D). In Imperial Hookah, significant amounts of sodium, lead, boron, iron, and chromium were also detected. All other elements/metals were found in lower amounts (Fig 4).

### Comparison of conventional cigarette smoke to EC/EH aerosols

The concentration of elements/metals in disposable EC/EH aerosol was compared to mainstream smoke from Marlboro Red cigarettes collected using ISO (Figs 2F and 4E) and CS protocols (Figs 2G and 4F). The total number of different elements found in smoke (N = 15) was less than the total number in EC/EH aerosol (N = 35). Cigarette smoke prepared using the ISO protocol yielded a higher total concentration of elements (4.259 μg/10 puffs) than most EC and EH aerosols, while smoke collected with the CS protocol had a lower total concentration (1.532 μg/10 puffs) than all ECs/EHs. As was seen with the ECs/EHs, silicon (ISO: 4.171, CS: 1.440 μg/10 puffs) was the dominant element in cigarette smoke. Lead, which was not detected in Marlboro Red cigarettes, was present in two brands of EHs with one having as high as 0.165 ± 0.048 μg/10 puffs.

### Effect of topography on elements in disposable EC/EH aerosol

**Fig 5:**
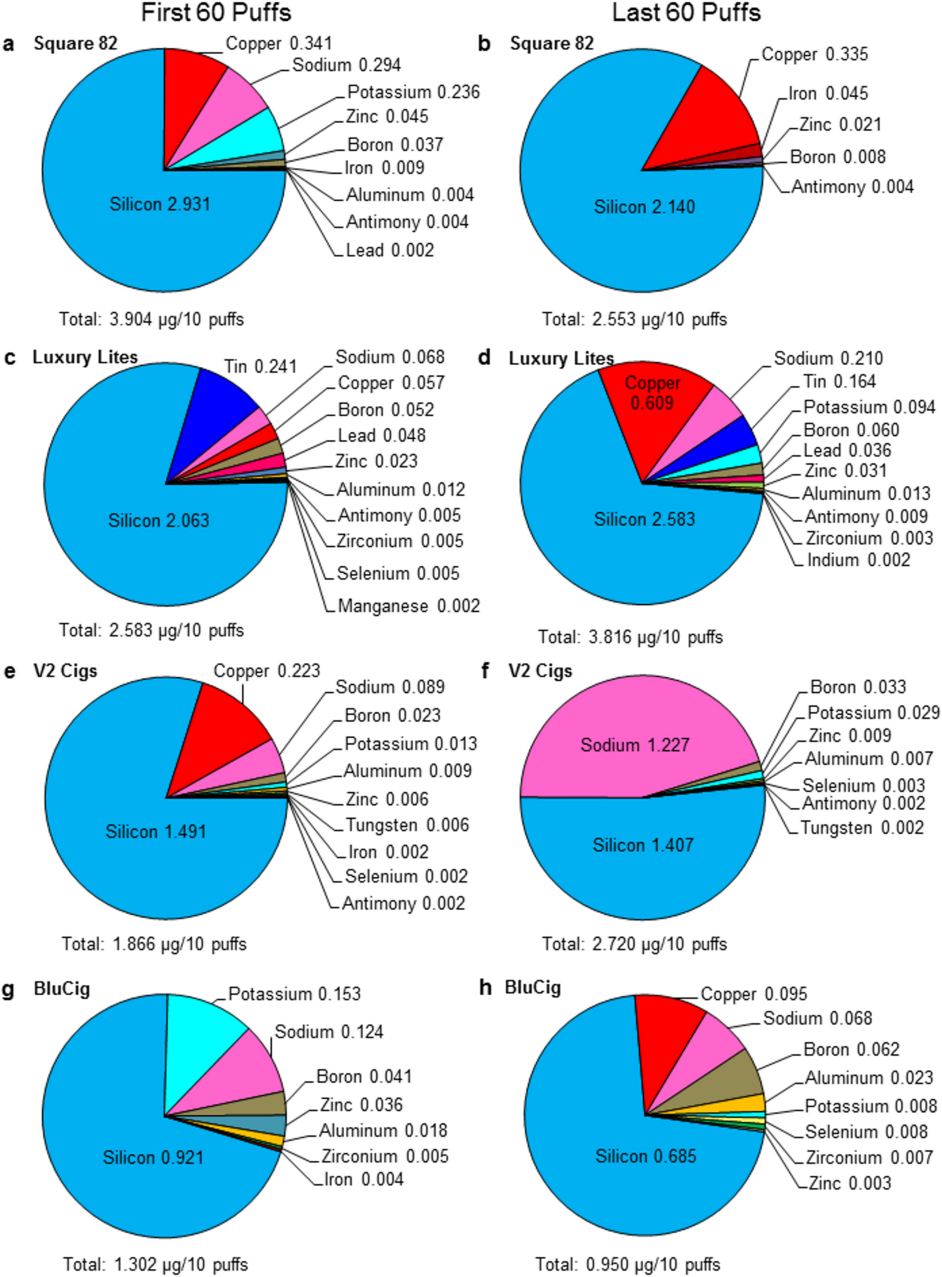
Comparison of the concentrations of elements in the first and last 60 puffs of disposable EC/EH aerosol. Four brands of disposable ECs/EHs were evaluated: (A, B) Square 82, (C, D) Luxury Lites, (E, F) V2 Cigs, and (G, H) BluCig. (A, C, E, G) Represent the concentration of elements in the first 60 puffs of each brand. (B, D, F, H) Represent the concentration of elements in the last 60 puffs of each brand. The total concentration of all elements is given at the bottom of the pie chart for each brand. Numbers adjacent to each element are concentrations in μg/10 puffs. All concentrations are the average of three independent aerosol samples from three different ECs/EHs, and only elements higher than or equal to 0.002 μg/10 puffs are presented in this figure.

To compare elements in aerosols created over time, the first 60 puffs and the last 60 puffs were generated using the lowest air-flow rate that produced robust aerosol from four brands of disposable ECs/EHs (Square 82, Luxury Lites, V2 Cigs, and BluCig) (Table 1). The concentration of elements/metals and their relative abundance in EC/EH aerosol are shown in Fig 5. The total concentration of elements/metals found in the first 60 puffs ranged from 1.302 to 3.904 μg/10 puffs (Fig 5A, 5C, 5E and 5G), with Square 82 having the highest overall total concentration and BluCig having the lowest (Fig 5A and 5G). The total concentration of elements/metals for the last 60 puffs ranged from 0.950 to 3.816 μg/10 puffs (Fig 5B, 5D, 5F and 5H) with Luxury Lites having the highest overall concentration and BluCig having the lowest concentration (Fig 5D and 5H). Silicon was the dominant element in both the first and last 60 puffs, and, for all brands except Luxury Lites, the concentration of silicon decreased during the last 60 puffs. Other metals detected in concentrations greater than 0.01 μg/10 puffs included copper, sodium, potassium, zinc, boron, iron, lead, and aluminum. Tin and lead were detected in Luxury Lites (Fig 5C and 5D). The relative amounts of the dominant elements (silicon and copper) were similar in Square 82 for the first and last 60 puffs. However, while silicon was always the dominant element in the remaining brands, the relative abundance of the other elements varied between the first and last 60 puffs. The most striking difference was seen in V2 cigs which had significantly more sodium in the last 60 puffs than in the first 60 puffs (Fig 5F).

### Comparison of individual elements across brands of disposable ECs/EHs and conventional cigarettes

**Fig 6:**
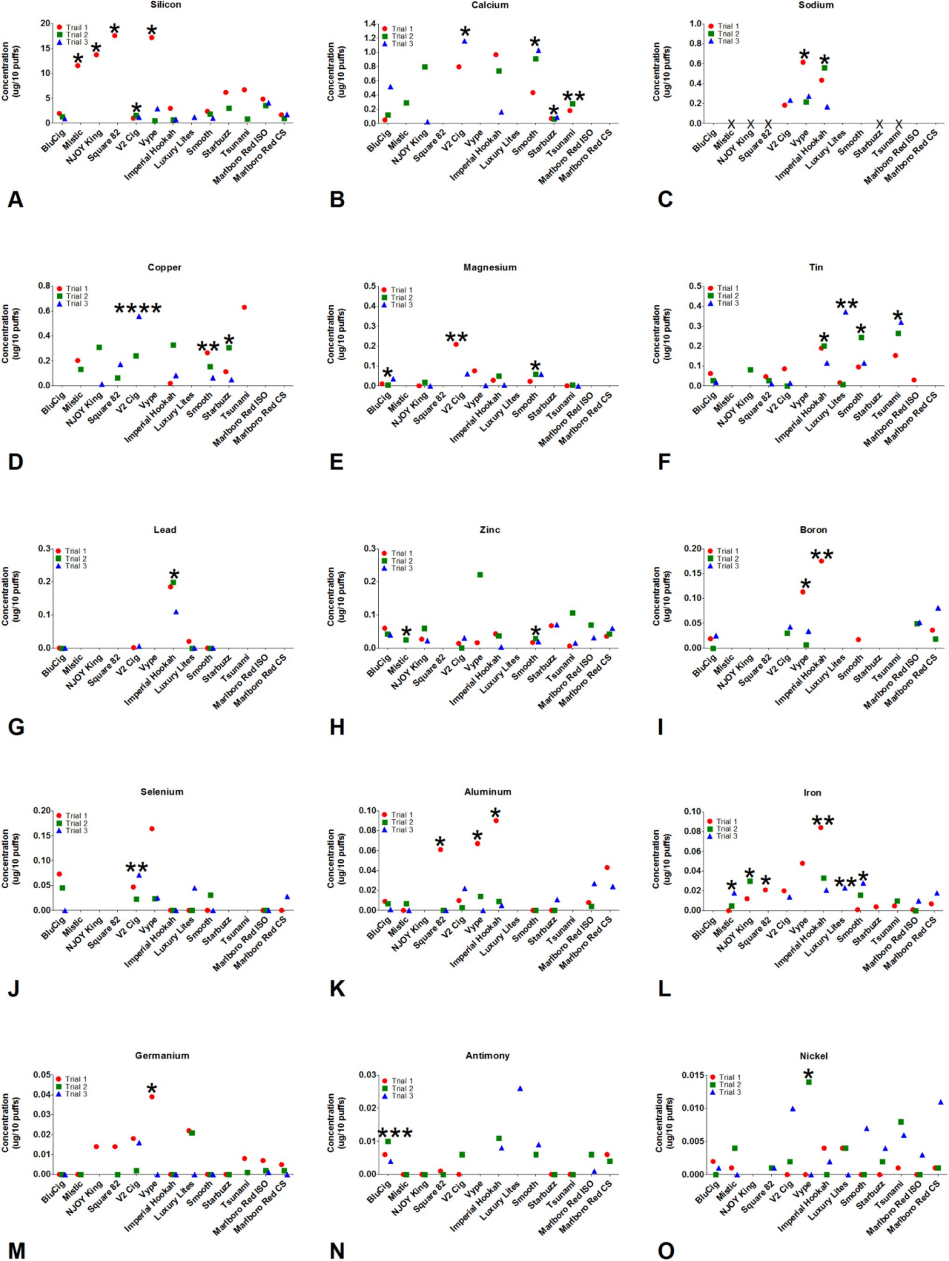
Comparison of individual elements across brands of disposable EC/EH aerosol and conventional cigarettes. The concentrations of 15 individual elements in EC/EH aerosol and Marlboro Red cigarette smoke (ISO and CS) are presented for each unit in each brand (A-O). Significant differences were evaluated by performing t-tests between the Marlboro Red group (ISO) and each individual EC or EH for each element. * = p < 0.05; ** = p < 0.01; *** = p < 0.001; **** = p < 0.0001. Absence of a dot indicates the value was below the limit of detection and the trial was treated as zero in the statistical analysis. Red = Trial 1, Green = Trial 2, Blue = Trial 3.

Individual elements in each product were compared to each other and to Marlboro Red (ISO and CS) to determine how element concentrations varied within and between brands (Fig 6). Significant differences were evaluated by performing t-tests between the Marlboro Red group (ISO) and each individual EC or EH for each element. Mercury was not found in any EC/EH product, while rubidium, arsenic, silver, cobalt, bismuth, palladium, and cadmium were rarely found (S1 Fig). Twelve of the elements (potassium, iridium, zirconium, tungsten, lanthanum, barium, indium, vanadium, chromium, molybdenum, manganese, titanium) were found in as few as four and as many as seven brands, but were not significantly different from the Marlboro Red group (S2 Fig). Sixteen of the elements (silicon, calcium, sodium, copper, magnesium, tin, lead, zinc, boron, selenium, aluminum, iron, germanium, antimony, nickel, strontium) were present in most of the brands of EC/EH, except for lead which was present in only two brands (Fig 6 and S3 Fig). In some brands, these elements were significantly higher than in the Marlboro Red group. The concentrations of elements were often variable both between brands and within EC/EH brands (Fig 6). In contrast, there was less variability in element concentrations in the Marlboro Red groups.

### Elemental analysis of the atomizer components in disposable ECs/EHs

**Fig 7:**
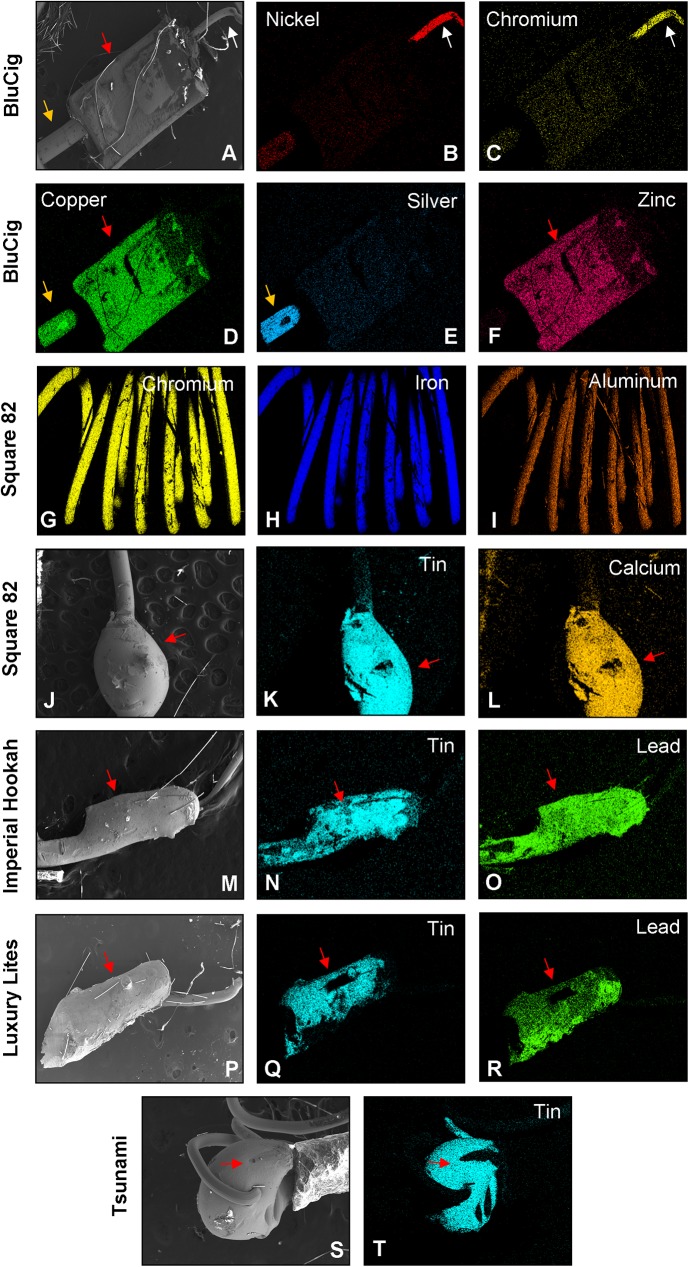
Scanning electron microscopy and energy dispersive X-ray spectroscopy analysis of disposable EC/EH wires and joints. (A) Scanning electron micrograph of the clamp joining thick and thin wires (red arrow) in BluCig. The filaments (0.13 mm) were usually comprised of nickel (B) and chromium (C) as shown for BluCig. For all brands, the thick wire (0.33 mm) was comprised of copper (D) and silver (E). The clamps in all brands were comprised of copper (D) and zinc (F) (2.4 mm). The filament (0.11 mm) from Square 82 was unusual in that it was comprised of chromium (G), iron (H), and aluminum (I). In some brands, the thick wire and filament were joined by tin solder. The solder joint (J) (1 mm) in Square 82 was comprised of tin (K) and calcium (L). The solder joint (M) (1.8 mm) between the thick wire and filament in Imperial Hookah was comprised of tin (N) and lead (O). The solder joint (P) (2 mm) between the thick wire and filament in Luxury Lites was comprised of tin (Q) and lead (R). (S) Example of poorly manufactured solder joints, comprised of tin (T) (0.78 mm) in most EC/EH brands. White arrow = filament (thin wire); Orange arrow = thick wire; Red arrow = joints between the thick and thin wires. Data are summarized in Table 2.

**Fig 8:**
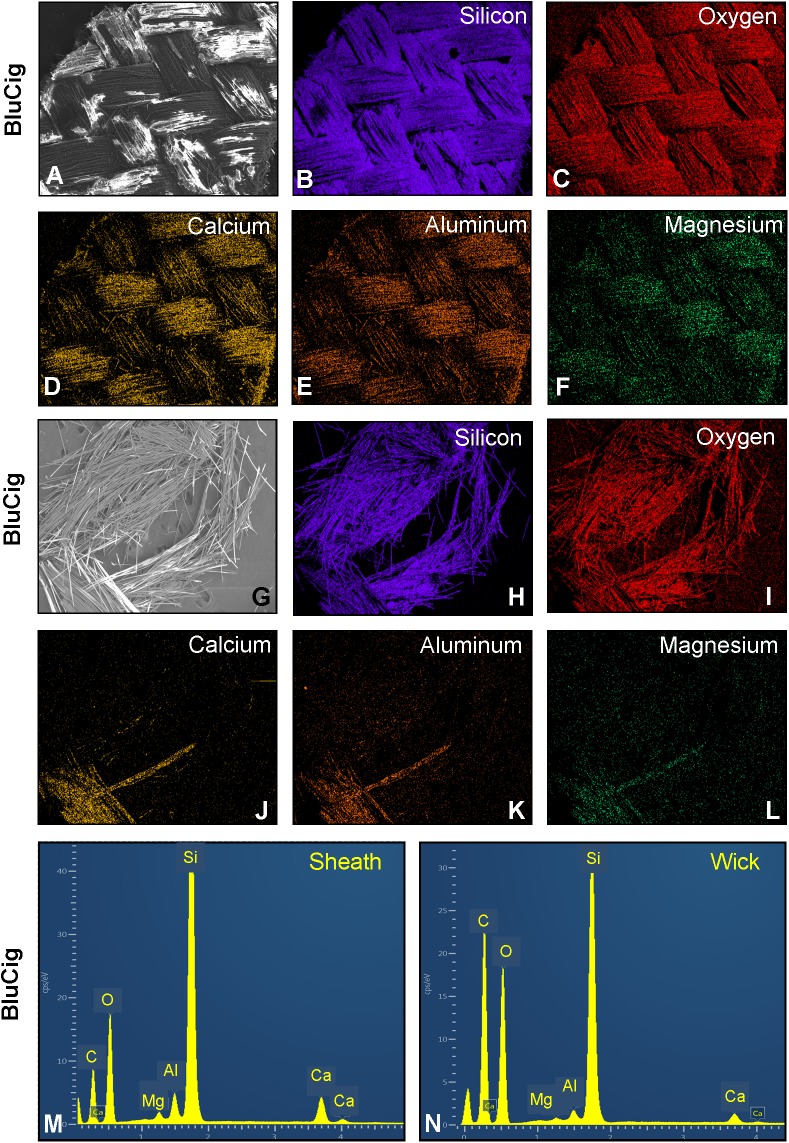
Scanning electron microscopy and energy dispersive X-ray spectroscopy analysis of disposable EC/EH wicks and sheaths. Examples are shown for BluCig which was representative of most brands. The sheath (A) in BluCig was comprised of silicon (B), oxygen (C), calcium (D), aluminum (E), and magnesium (F). For all brands, the wicks all had a similar composition as shown for BluCig. The wick (G) was comprised of silicon (H), oxygen (I), calcium (J), aluminum (K), and magnesium (L). Spectra of the composition of the sheath (M) and wick (N) are presented in this figure. All data are summarized in Table 2.

To determine the elemental composition of the components of disposable ECs/EHs, dissected atomizer units were examined using SEM (Figs 7 and 8, Table 2 and S4 Fig). The structure and composition of the filament, thick wire, and joints between wires are shown for BluCig in Fig 7. For most brands (BluCig, NJOY King, Mistic, V2 Cigs, Luxury Lites, Smooth, Tsunami, and Imperial Hookah), the filament was comprised of nickel and chromium (Fig 7B and 7C, S4A Fig and Table 2). In contrast, filaments in Square 82 contained mainly chromium, iron, and aluminum (Kanthal) (Fig 7G–7I), as well as molybdenum, titanium, and copper (S4B Fig). Vype and Starbuzz had iron, chromium, and nickel in the filament (Table 2).

**Table 2:** Elemental composition of the atomizer of EC/EH products.

Brand	Thin Filament	Thick Wire	Wire to Wire Joint	Wire to Battery Joint	Wick	Sheath
BluCig	Chromium,Nickel	Copper, Silver Coated	Copper, Zinc Clamp	Tin Solder	Silicon, Oxygen and Silicon, Oxygen, Magnesium, Calcium, Aluminum	Silicon, Oxygen, Magnesium, Calcium, Aluminum
Mistic	Chromium,Nickel^a^	Copper, Silver Coated^a^	Copper, Zinc Clamp^a^	Tin Solder^a^	Silicon, Oxygen, Magnesium, Calcium, Aluminum	Silicon, Oxygen, Magnesium, Calcium, Aluminum
NJOY King	Chromium,Nickel	Copper, Nickel, Silver Coated	Copper,Zinc Clamp	Tin Solder	Silicon, Oxygen	Silicon, Oxygen, Calcium^b^
Square 82	Chromium, Copper, Aluminum, Titanium, Molybdenum, Iron	Copper, Silver Coated	Tin, Calcium Solder	Tin Solder	Silicon, Oxygen	Silicon, Oxygen, Magnesium, Calcium, Aluminum
V2 Cigs	Chromium,Nickel	Copper, Silver Coated	Tin Solder	Tin Solder	Silicon, Oxygen	Silicon, Oxygen, Magnesium, Calcium, Aluminum
Vype	Chromium,Nickel, Iron	Copper, Silver Coated	Copper, Zinc Clamp	Tin Solder	Silicon, Oxygen	Silicon, Oxygen, Magnesium, Calcium, Aluminum,
Imperial Hookah	Chromium,Nickel	Copper, Silver Coated	Tin, Lead Solder	Tin, Lead Solder and Organic glue	Silicon, Oxygen	Silicon, Oxygen, Magnesium, Calcium, Aluminum
Luxury Lites	Chromium,Nickel	Copper, Silver Coated	Tin, Lead Solder	Tin, Lead Solder	Silicon, Oxygen, Calcium, Aluminum	Silicon, Oxygen, Magnesium, Calcium, Aluminum, Sodium
Smooth	Chromium, Nickel	Copper, Tin Coated	Tin Solder	Tin Solder	Silicon, Oxygen	Silicon, Oxygen, Magnesium, Calcium, Aluminum, Sodium
Starbuzz	Chromium, Nickel, Iron	Copper, Nickel, Silver Coated	Copper, Zinc Clamp	Tin Solder	Silicon, Oxygen	Silicon, Oxygen, Magnesium, Calcium, Aluminum
Tsunami	Chromium,Nickel	Copper, Nickel, Silver Coated	Tin Solder	Tin Solder	Silicon, Oxygen	Silicon, Oxygen, Magnesium, Calcium, Aluminum

^a^Data presented in Williams et al 2015 PlosOne.

^b^Elemental maps of silicon, oxygen, and calcium were generated, magnesium and aluminum were also in the spectrum.

The thick wire was usually made of copper coated with silver as shown for BluCig (Fig 7D–7E). In contrast, NJOY King, Tsunami, and Starbuzz had a copper and nickel thick wire coated with silver (S4F Fig and Table 2), and Smooth had a copper wire coated with tin (Table 2). The thick wire and filament were joined using either clamps or solder. Five brands (BluCIg, NJOY King, Mistic, Vype, and Starbuzz) had wires joined by copper/zinc (brass) clamps (Fig 7A, 7D and 7F, S4A Fig and Table 2), while the remaining brands (Square 82, V2 Cigs, Luxury Lites, Smooth, Tsunami, and Imperial Hookah) had wires joined with solder (Fig 7J, S4C and S4F Fig and Table 2).

The solder in all brands was predominantly made of tin as shown for Square 82 and Tsunami (Fig 7J, 7K, 7S and 7T). The tin solder in Square 82 also contained calcium (Fig 7L). Imperial Hookah and Luxury Lites had both tin and lead in the solder joint between wires (Fig 7M–7R and Table 2) and lead was found in their aerosols (Figs 4A, 5C and 5D). While the joints between the thick wire and battery were made of tin solder in all brands, these joints in Luxury Lites and Imperial Hookah also contained lead (Table 2).

In all brands of EC/EH, the sheaths, which are added for insulation, were comprised of silicon, oxygen, calcium, aluminum, and magnesium with silicon being the dominant element as shown for BluCig (Fig 8A–8F and 8M and Table 2). Luxury Lites and Smooth had sodium in addition to the above mentioned elements in their sheaths (Table 2). NJOY King was the only brand for which the sheath was comprised mainly of silicon, oxygen, and calcium (Table 2).

The wick, which the filament wraps around, was predominantly silicon and oxygen, as shown for BluCig (Fig 8G–8I, and Table 2). For BluCig, Mistic and Luxury Lites, the wicks had the same composition as the sheath, with Luxury Lites lacking magnesium (Fig 8J–8L and 8N and Table 2).

## Discussion

This is the first study to analyze elements/metals in the aerosols from disposable ECs/EHs. Thirty-five elements/metals were found in aerosols of 11 different brands of ECs/EHs (Table 3). Vype and Square 82 had the highest total concentration of elements/metals, while Mistic and BluCig had the lowest. Twenty-one of the elements found in EC/EH aerosol (e.g. copper, tin, and lead) were not present in conventional cigarette smoke, while others (e.g. zinc, aluminum and iron) were present in both aerosol and smoke. Fifteen of the 21 elements (calcium, sodium, copper, magnesium, tin, lead, zinc, boron, aluminum, iron, germanium, antimony, selenium, nickel and strontium) were found in most EC/EH products, in some cases at concentrations that were significantly higher than in conventional cigarettes. Silicon was the dominant element in all aerosols from EC/EH products as well as in smoke from conventional cigarettes. In two brands of EHs, lead was present in both the tin solder joints and aerosol. The total concentration of elements was higher at low air-flow rates, while the number of individual elements was similar at both low and high air-flow rates. However, the composition of the elements in aerosols was different in the first and last 60 puffs.

**Table 3:** Frequency of an element present in EC/EH aerosol.

Element	# of Brands in Which Element Was Not Not Detected		# of Brands in Which Element Was Detected at 0.001 to 0.01 μg/10 puffs		# of Brands in Which Element Was Detected at >0.01 μg/10 puffs	
	E-Cigarette	E-Hookah	E-Cigarette	E-Hookah	E-Cigarette	E-Hookah
Aluminum	1	3	2	1	3	1
Antimony	4	3	2	2		
Arsenic	5	4	1	1		
Barium	2	4	4	1		
Bismuth	6	5				
Boron	3	3		1	3	1
Cadmium	6	5				
Calcium	2	1			4	4
Chromium	6	3		2		
Cobalt	6	4		1		
Copper	2	1	1		3	4
Germanium	4	4	1		1	1
Indium	5	4	1	1		
Iridium	6	5				
Iron	2	1	3	2	1	2
Lanthanum	5	3	1	2		
Lead	6	3		1		1
Magnesium	2	2	2	1	2	2
Manganese	5	3	1	2		
Mercury	6	4		1		
Molybdenum	4	5	2			
Nickel	2		4	5		
Palladium	6	5				
Potassium	2	2			4	3
Rubidium	5	4	1			1
Selenium	3	3		1	3	1
Silicon					6	5
Silver	5	5	1			
^a^Sodium	2	1	1		1	1
Strontium	2	1	4	4		
Tin	2	1			4	4
Titanium	6	5				
Tungsten	5	3		2	1	
Vanadium	3	4	3	1		
Zinc	1	1	1		4	4
Zirconium	2	2	2	1	2	2

^a^Sodium was only screened in six brands of EC/EH.

The concentrations of specific elements in Marlboro Red smoke were usually similar for different samples, and a similar relationship was also observed for some of the EC/EH products. For example, the variance for specific elements was small for each individual BluCig unit. However, the concentrations of specific elements within brands of EC/EH was sometimes highly variable (e.g., silicon in Mistic, NJOY King, Square 82 and Vype or tin in Luxury Lites). Users of EC/EH products, who do not normally have a method to identify ECs/EHs with high concentrations of elements/metals, should be aware of these variations in concentrations between and within brands. Moreover, EC users have highly variable topographies [16] which could also affect concentrations of elements/metals in aerosol and our numbers may underestimate concentrations for some users. Improvements in manufacturing and design could make element/metal emissions in EC/EH aerosols more uniform within a brand and reduce those that are relatively high in concentration.

While the external features of disposable ECs/EHs differed in diameter, length, and shell design [1], the components of the atomizing chambers were similar among brands. The overall anatomy of the atomizers was also similar to that of cartomizer ECs, except that the sheath covering the filament was much longer in the disposable brands [3]. Button-activated models had a circuit board outside the atomizing chamber that could contribute to elements in the aerosol.

Mercury was not found in any of the EC/EH aerosols, while rubidium, arsenic, silver, cobalt, bismuth, palladium, and cadmium were found in only one or two samples of aerosol (Table 3). The results with mercury would need confirmation since it is difficult to analyze by ICP-OES [17]. In a prior study, up to 0.22 μg/150 puffs of cadmium were detected in the aerosol of other EC brands [4]. This discrepancy in the cadmium data may be due to differences in the brands used, number of puffs collected, and/or the methods of analysis. The silver found in one aerosol likely came from the silver coating on the thick wire. Because silver has a high melting point (962°C) and the thick wire was always covered by Teflon, silver may not pass readily from the wire into the aerosol.

Twelve elements (potassium, iridium, zirconium, tungsten, lanthanum, barium, indium, vanadium, chromium, molybdenum, manganese, titanium), which were not significantly different in concentration from the Marlboro Red group (ISO), were each present in at least four brands of EC/EH (S2 Fig). The lack of significant difference may be due to the large variances that were seen between units within some brands (e.g., chromium and lanthanum in Smooth). In all brands, the filament was made of chromium and nickel (nickel concentrations were sometimes significantly higher than in cigarette smoke), which were present in low levels in aerosols, perhaps due to their relatively high melting points (1857°C and 1453°C, respectively). Our data are consistent with concentrations of chromium and nickel reported previously in EC aerosols [2,4]. Molybdenum and titanium were present in the filament of Square 82, but not its aerosol, suggesting the metal alloy is stable during heating cycles. The sources of the other elements in S2 Fig were not identified by SEM/EDS, either because their levels were below the limit of detection for the SEM/EDS or because they originated outside of the atomizing chamber, which was the only part of the EC/EH analyzed.

Sixteen elements (silicon, calcium, sodium, copper, magnesium, tin, lead, zinc, boron, selenium, aluminum, iron, germanium, antimony, nickel and strontium) were significantly different in concentration from the Marlboro Red group (ISO) and were present in most brands of EC/EH (Fig 6 and S3 Fig). All elements in this group were present in at least one brand of EC/EH at concentrations significantly higher than in the Marlboro Red group, and these will be discussed in more detail.

Silicon was the most abundant element in the aerosols of all brands, regardless of topography, and in most samples, accounted for over 50% of the total weight of all the elements (Fig 6). Its concentrations (0.094 to 6.835 μg/10 puffs) overlapped the 2.24 μg/10 puffs reported previously in a cartomizer style EC [2]. The most likely sources of the aerosolized silicon were the sheath and wick, which were made of delicate finely woven silicate glass threads with minor sodium, potassium, calcium and magnesium (fiberglass) that can easily break when handled. The silicon in the aerosol probably came from fragments of the sheath/wick that broke off during manufacturing and/or are released during heating/cooling cycles. Consistent with the latter idea, some wicks were blackened and damaged near the filament after use (not shown). We previously observed small silicate beads that may have included nanoparticles on the surface of the wick and in aerosols from a cartomizer EC [2]. Further work is needed on the forms of silicon and silicate fiberglass delivered by ECs/EHs and their health effects associated with their inhalation.

Copper (0.044 to 0.610 μg/10 puffs), which was one of the most abundant elements in the aerosol of most brands of EC/EH (Fig 6), was similar in concentration to those reported in other cartomizer style ECs (0.011 to 0.203 μg/10 puffs) [2,3], but not as high as the concentrations (0.243 to 2.247 μg/10 puffs) found by Lerner et al 2014. The most likely sources of copper were the thick wire and brass clamps. Although copper has a high melting point (1084°C), it is possible that with rapid heating, high air-flow, and frequent puffs, the thick wire and brass clamps, which are in close proximity to the filament, emit copper into the aerosol. EC aerosols contain copper nanoparticles [18], and it is likely that some of the mass from the copper we detected was due to nanoparticles. The inhalation of copper nanoparticles can cause damage to the kidney, liver, and spleen [19], and long-term inhalation of copper could lead to nose, skin, and eye irritation as well as headaches, dizziness, and nausea [20,21], which have been reported by EC users [7,22].

Tin (0.016 to 0.245 μg/10 puffs) was found in 9 of 11 brands of EC/EH aerosol, and its concentrations were significantly higher than in Marlboro Red smoke in four of the five EH brands (Fig 6). None of the ECs/EHs had concentrations as high as 11.368 μg/10 puffs, which we observed previously in a cartomizer style EC [3]. Tin was detected in the aerosol of the six brands of EC/EH that used tin solder joints between the filament and thick wire. Because tin melts at 232°C and ECs can heat to over 300°C [23], it is probable that melting solder joints contributed tin to the aerosol. In support of this, the concentration of tin was highest in Imperial Hookah, Luxury Lites, Smooth, and Tsunami, which all had friable solder joints that did not cover the underlying wires. Three brands (BluCig, NJOY King, and Vype) used brass clamps between the wires, and the tin in their aerosol likely originated from the solder joints between the wire and battery. Square 82 was the only brand with solder joints containing tin and calcium; in this brand, there was no calcium detected in the aerosol, and the solder joints appeared intact by SEM, which agrees with the concentration of tin being low. Inhalation of tin can cause respiratory irritation and prolonged exposure can result in stannosis [8,24,25].

Lead was present in the solder joints and aerosol of Imperial Hookah and Luxury Lites (0.007 to 0.165 μg/10 puffs) (Fig 6). The concentration in Imperial Hookah and Luxury Lites were higher than that reported previously in several ECs [2,4]. Historically, lead has been used to stabilize solder. However, because of its toxicity, the use of lead in solder has been banned in many countries including China, where most EC/EH products are manufactured [26]. Our data show that the ban on using lead in solder is not strictly enforced, and EC/EH users cannot assume their products are lead free. Lead has a melting point of 327°C, which is within the heating range of ECs/EHs [23]. Ironically, in most of the Imperial Hookah and Luxury Lites solder joints, the solder appeared to have partially melted, indicating that lead was not helpful in stabilizing these joints. Products from both Imperial Hookah and Luxury Lites were tested in 2013 and again in 2015, and the solder joints from both purchases contained lead. Inhaled lead can be absorbed in the respiratory tract and distributed to the soft tissues, liver, and central nervous system [27]. Lead is classified as a carcinogen by the Federal Drug Administration (FDA) and Environmental Protection Agency (EPA) [10, 26–30].

Three alkali and alkaline earth metals (sodium, magnesium, and calcium) were frequently present in the aerosols of disposable ECs/EHs (Fig 6). Disposable ECs delivered higher concentrations of these elements than a cartomizer style EC [2]. The sheath and wick were the likely sources of the magnesium, calcium, and sodium. These elements occurred as oxides or silicates and their melting temperatures would have been above 1000°C. These elements could cause mild to moderate skin and eye irritation, and chronic inhalation could lead to liquefaction necrosis of soft tissues [31], which might explain why some users have reported pain in muscles, as well as skin and eye complications [7].

Zinc (0.003 to 0.048 μg/10 puffs) was detected in the aerosol of all 11 brands of EC/EH (Fig 6), but was lower in concentration than we previously detected in aerosols from cartomizer EC (0.127 μg/10 puffs) [3]. The major source of zinc was likely the brass clamps that joined the filament and the thick wire. Five brands with brass clamps (BluCig, NJOY King, Mistic, Vype, and Starbuzz) had higher zinc concentrations in their aerosols than brands with solder between the thick and thin wires. Although all brands contained zinc in their aerosols, 6 of 11 brands did not contain brass clamps, indicating that zinc could come from other sources, such as the shell, circuit board, or the battery chamber, which were not analyzed by SEM/EDS. Zinc has a relatively low melting point (420°C), which could explain its presence in the aerosols. In most ECs/EHs, zinc concentrations were similar within and between brands and similar also to zinc concentrations in Marlboro Red smoke (ISO and CS). Inhalation of zinc could contribute to metal fume fever, decreased pulmonary function [8,32], chest pains, and coughing, which are symptoms that EC users have reported [7].

Eight elements (boron, selenium, aluminum, iron, germanium, antimony, nickel and strontium) (Fig 6 and S3 Fig) were overall lower in concentration than the above-mentioned elements, but each of these elements was significantly higher in concentration in 1 to 6 of the EC/EH aerosols than in the Marlboro Red cigarette smoke. The source of the aluminum is most likely the sheath, and the source of iron is unknown, but is likely the shell that encloses the atomizing unit as previously reported in a cartomizer style EC [2]. Nickel as mentioned before was predominantly found in the thin nichrome filament of the atomizing unit. The sources of the other elements could not be determined by SEM/EDS, probably because their concentrations were below the limit of detection for this method. Inhalation of these elements can deposit in the lung and cause respiratory irritation [33–35].

The Marlboro Red cigarettes that were puffed according to the ISO protocol had a higher total concentration of elements than the cigarettes smoked using the CS protocol and all EC/EH brands except Vype and Square 82. The lower concentration of elements in the smoke from the CS protocol may be due to the higher air-flow rate used, which diluted the concentration of elements [36], a pattern that was also seen with the NJOY King EC puffed at low and high air-flow rates. It is also possible that the elements differed in the tobacco used for ISO and CS smoke, since the packs were purchased at different times, and metals in conventional cigarette smoke vary with origin of the tobacco leaves [37,38]. The total number of elements was greater in all ECs/EHs than in the Marlboro Reds smoked using the ISO and the CS protocols. Of 36 elements screened, 21 (calcium, sodium, copper, magnesium, lead, potassium, strontium, selenium, rubidium, arsenic, silver, cobalt, bismuth, palladium, cadmium, iridium, tungsten, lanthanum, vanadium, molybdenum, manganese) were detected in at least one brand of EC/EH, but not in Marlboro Red smoke. Chromium, lead, and manganese have been reported in cigarette smoke by others [39–42], which again may be related to differences in tobacco leaves [40,41].

Our data will be useful in future assessments of risk associated with inhalation of elements/metals in EC/EH aerosols. ECs/EHs are recreational products that produce complex mixtures of elements/metals in aerosols by heating fluid and metal components in the atomizing unit. Most regulatory standards, such as minimal risk levels (MRL), permissible exposure limits (PEL), or recommended exposure limits (REL), are intended for occupational, not recreational, exposure. Occupational limits are normally established for single elements in ambient air and do not take into account the complex mixtures of elements/metals that are produced during heating of ECs/EHs. Moreover, occupational limits are recommended to not exceed inhalation of a particular element over the course of a normal work week, while ECs/EHs are recreational products that consumers inhale aerosol intermittently throughout the day, making their exposure pattern different than occupational exposure.

The most appropriate method for evaluating risk from elemental/metal exposure of inhaled EC/EH aerosol would be an EPA risk cup assessment that takes into account all elements present in an aerosol. The EPA’s “risk cup”, an addition to the Food Quality Protection Act (FQPA), was created to assess cumulative exposures (diet, air, water, non-occupational, etc.) to pesticides and establish maximum permissible exposures for all constituents from a group of chemicals in contrast to assessing the risk of an individual chemical [43–45]. When the risk cup is full (exceeding 100%) no further exposure is permitted and regulatory attention is needed [44]. Currently, this type of assessment cannot be performed on EC/EH aerosols as there is not acute, sub-chronic, and chronic inhalation data for most elements/metals in EC/EH aerosols, and therefore the appropriate reference concentrations, which are need for performing a risk cup assessment, have not been established for EC/EH metal exposures. Our study does identify the types and concentrations of elements in EC/EH aerosol and provides foundational information on which elements should be evaluated in the future to acquire inhalation reference concentrations. We did a preliminary risk cup assessment (not shown) using available reference doses (not concentrations) for oral exposure of some of the elements/metals in EC/EH aerosols and found that selenium and antimony in many of the brands exceeded the 100% risk cup limit. This preliminary observation emphasizes the need for reference concentrations for the elements reported in EC/EH aerosols so that in the future, a risk cup assessment can be performed using reference concentrations for all elements in these aerosols.

In summary, our results demonstrate that aerosols from popular disposable ECs/EHs contain at least 35 elements/metals, 21 of which were not found in cigarette smoke. Some elements/metals were present in significantly higher concentrations in EC/EH aerosol than in cigarette smoke. There is variability between and within brands in element concentration and in the number of elements present in EC/EH aerosol. Most elements/metals in EC/EH aerosols likely originated from components in the atomizer, such as the filament, solder joints, wick and sheath. In evaluating the potential health effects of the elements/metals in EC/EH aerosols, it will be important to consider that they are inhaled as a complex mixture which is heated, and it will be necessary to have more information on reference concentrations for a valid risk cup assessment of the elements/metals detected in EC/EH aerosols.

## Supporting information

S1 Material Additional materials and methods. (DOCX)

S1 Table Limits of quantification and melting points of analytes analyzed. (DOCX)

S1 Fig Comparison of individual elements rarely detected in brands of disposable EC/EH aerosol and conventional cigarettes. The concentrations of eight individual elements that were rarely detected in EC/EH aerosol and not detected in Marlboro Red cigarette smoke (ISO and CS) are presented for each trial in each brand (A-H). None of the elements in EC/EH aerosol was significantly different than the Marlboro Red (ISO) group. Absence of a dot indicates the value was below the limit of detection and the trial was treated as zero in the statistical analysis. Red = Trial 1, Green = Trial 2, Blue = Trial 3.
(TIF)

S2 Fig Comparison of individual elements across brands of disposable EC/EH aerosols and conventional cigarettes. The concentrations of 12 individual elements in EC/EH aerosol and Marlboro Red cigarette smoke (ISO and CS) are presented for each trial in each brand (A-l). Elements in this figure were frequently detected in EC/EH aerosol, but concentrations were not significantly different than in the Marlboro Red (ISO). Absence of a dot indicates the value was below the limit of detection and the trial was treated as zero in the statistical analysis. Red = Trial 1, Green = Trial 2, Blue = Trial 3.
(TIF)

S3 Fig Comparison of concentration of strontium across brands of disposable EC/EH aerosol and conventional cigarettes. The concentrations of strontium in EC/EH aerosol and Marlboro Red cigarette smoke (ISO and CS) are presented for each trial in each brand. The concentration of strontium was significantly higher in Smooth EH than in Marlboro Red (ISO). * = p < 0.05. Red = Trial 1, Green = Trial 2, Blue = Trial 3.
(TIF)

S4 Fig Spectral data from the energy dispersive X-ray spectroscopy analysis of components presented in Fig 6. (A) BluCig thick wire to filament joint, (B) Square 82 filament, (C) Square 82 solder joint, (D) Imperial Hookah solder joint, (E) Luxury Lites solder joint, and (F) Tsunami solder joint.
(TIF)
