# Supplementary material for: Elements including metals in the atomizer and aerosol of disposable electronic cigarettes and electronic hookahs
Source: PLoS One. 2017 Apr 17;12(4):e0175430. doi: 10.1371/journal.pone.0175430 (PMC5393578; doi:10.1371/journal.pone.0175430)
Supplement: S1 Table — (DOCX) [file pone.0175430.s002.docx]

**Supplemental Table 1. Limits of Quantification and Melting Points of Analytes Analyzed.**

| **Analyte Name (Abbreviation)** | **Element**  **Classification** | **Limit of Quantification**  **(mg/L)** | **Melting Temperature (°C)** |
| --- | --- | --- | --- |
| Ag | Transition | 0.002 | 962 |
| Al | Post-Transition | 0.004 | 660 |
| As | Metalloid | 0.008 | 81 |
| B | Metalloid | 0.003 | 2300 |
| Ba | Alkaline Earth | 0.000 | 725 |
| Bi | Post-Transition | 0.011 | 271 |
| Ca | Alkaline Earth | 0.003 | 839 |
| Cd | Transition | 0.001 | 321 |
| Cr | Transition | 0.002 | 1857 |
| Co | Transition | 0.001 | 1495 |
| Cu | Transition | 0.002 | 1083 |
| Fe | Transition | 0.001 | 1535 |
| Ge | Metalloid | 0.009 | 937 |
| In | Post-Transition | 0.007 | 157 |
| Ir | Transition | 0.242 | 2410 |
| K | Alkali | 0.002 | 64 |
| La | Lanthanide | 0.001 | 920 |
| Mg | Alkaline Earth | 0.000 | 639 |
| Mn | Transition | 0.000 | 1245 |
| Mo | Transition | 0.002 | 2617 |
| Na | Alkali | 0.001 | 98 |
| Ni | Transition | 0.001 | 1453 |
| Pb | Post-Transition | 0.007 | 327 |
| Pd | Transition | 0.008 | 1552 |
| Rb | Alkali | 0.004 | 39 |
| Se | Nonmetal | 0.017 | 217 |
| Si | Metalloid | 0.003 | 1410 |
| Sn | Post-Transition | 0.004 | 232 |
| Sr | Alkaline Earth | 0.000 | 769 |
| Ti | Transition | 0.000 | 839 |
| V | Transition | 0.001 | 1890 |
| W | Transition | 0.003 | 3410 |
| Zn | Transition | 0.001 | 420 |
| Zr | Transition | 0.001 | 1852 |
