# Supplementary figures and images for: Elements including metals in the atomizer and aerosol of disposable electronic cigarettes and electronic hookahs
Source: PLoS One. 2017 Apr 17;12(4):e0175430. doi: 10.1371/journal.pone.0175430 (PMC5393578; doi:10.1371/journal.pone.0175430)

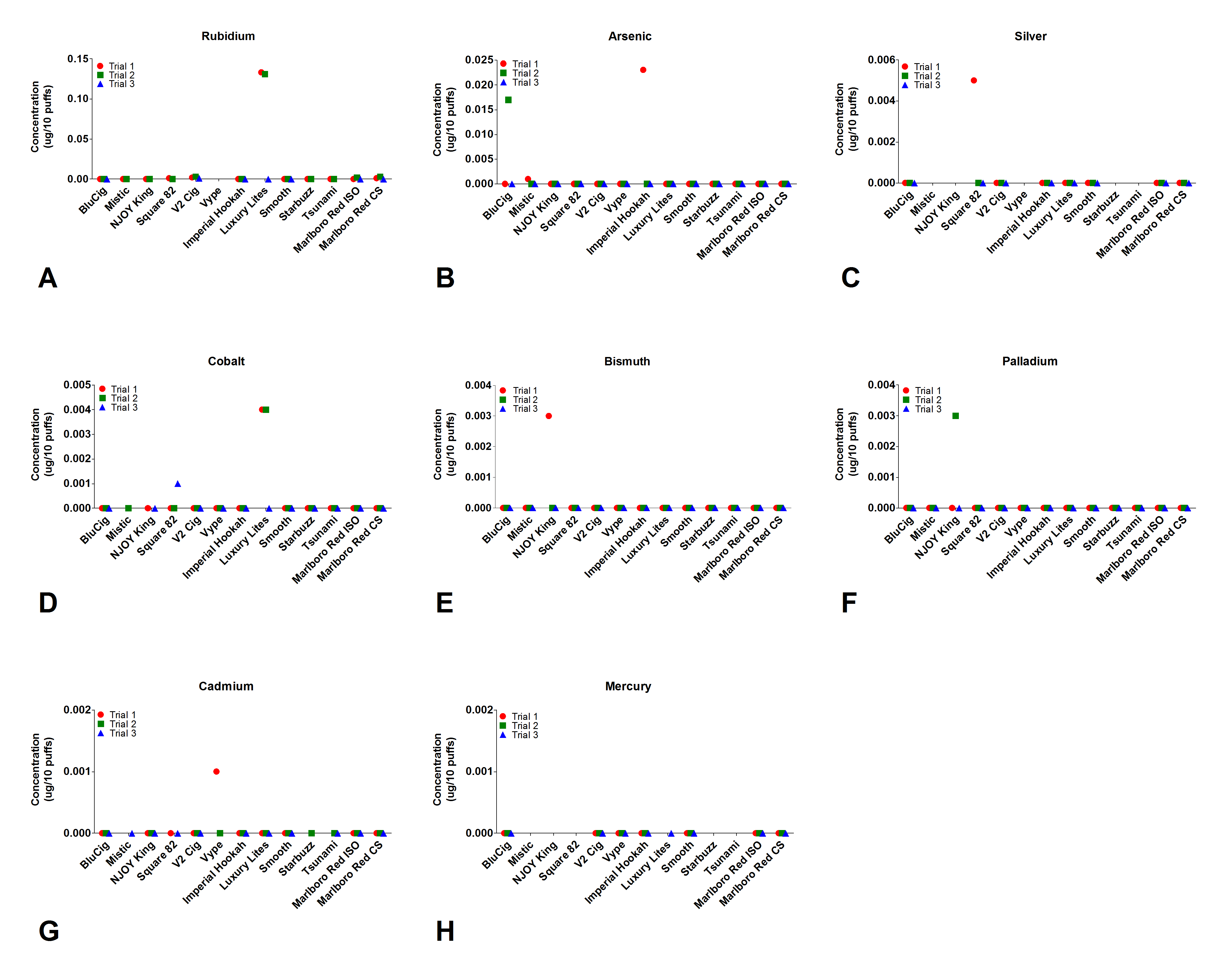

Supplement: S1 Fig — The concentrations of eight individual elements that were rarely detected in EC/EH aerosol and not detected in Marlboro Red cigarette smoke (ISO and CS) are presented for each trial in each brand (A-H). None of the elements in EC/EH aerosol was significantly different than the Marlboro Red (ISO) group. Absence of a dot indicates the value was below the limit of detection and the trial was treated as zero in the statistical analysis. Red = Trial 1, Green = Trial 2, Blue = Trial 3. (TIF) [file pone.0175430.s003.tif]

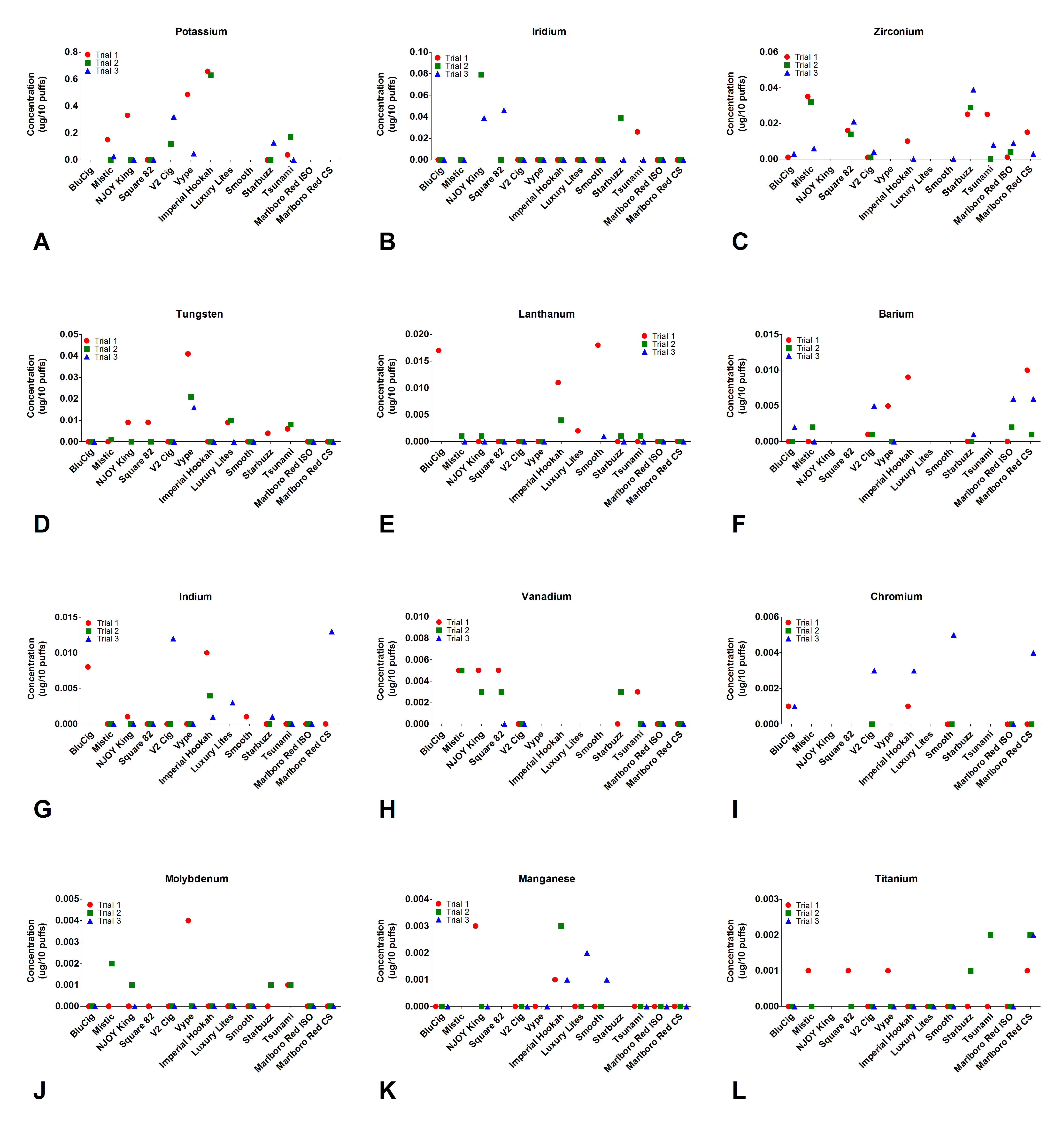

Supplement: S2 Fig — The concentrations of 12 individual elements in EC/EH aerosol and Marlboro Red cigarette smoke (ISO and CS) are presented for each trial in each brand (A-l). Elements in this figure were frequently detected in EC/EH aerosol, but concentrations were not significantly different than in the Marlboro Red (ISO). Absence of a dot indicates the value was below the limit of detection and the trial was treated as zero in the statistical analysis. Red = Trial 1, Green = Trial 2, Blue = Trial 3. (TIF) [file pone.0175430.s004.tif]

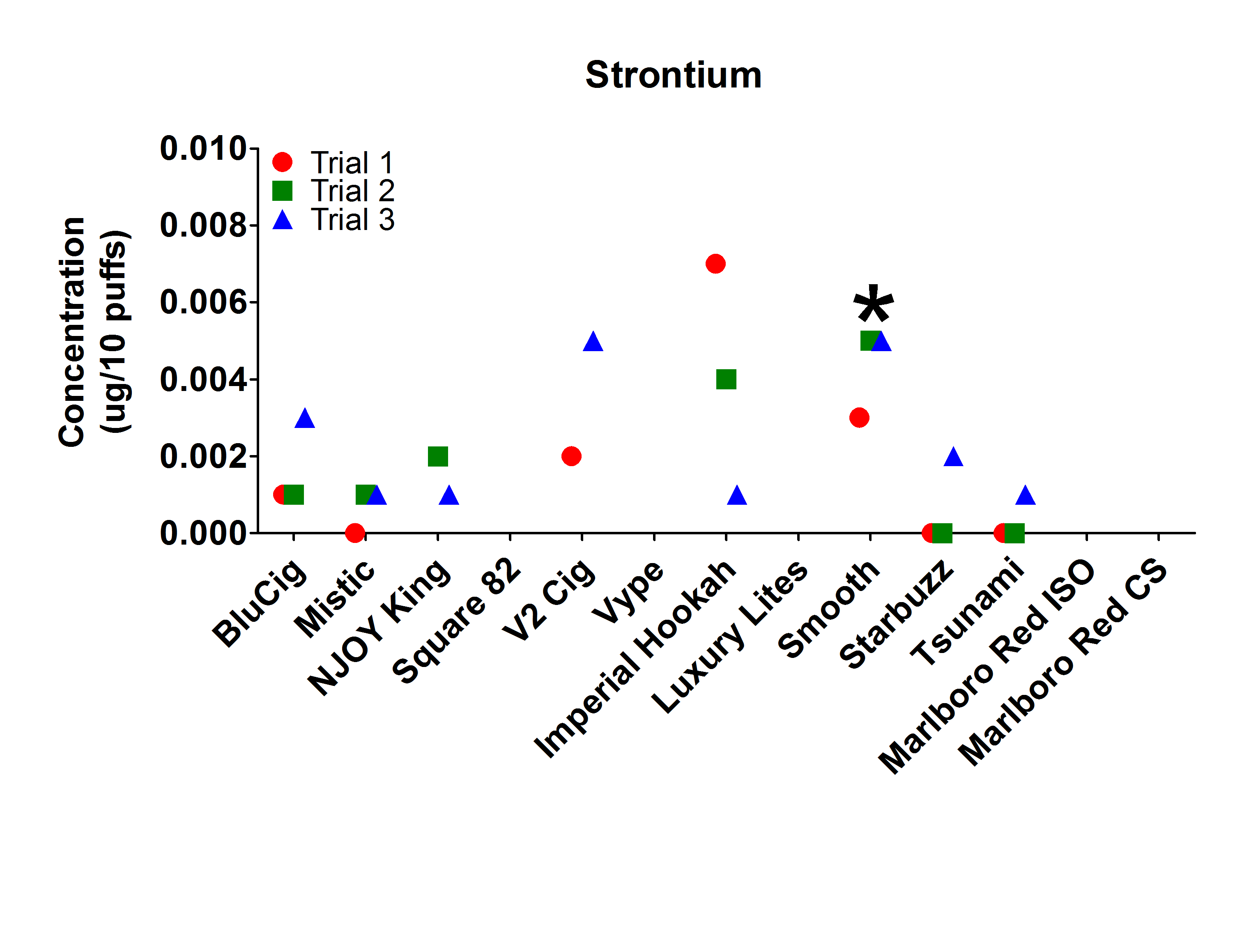

Supplement: S3 Fig — The concentrations of strontium in EC/EH aerosol and Marlboro Red cigarette smoke (ISO and CS) are presented for each trial in each brand. The concentration of strontium was significantly higher in Smooth EH than in Marlboro Red (ISO). * = p < 0.05. Red = Trial 1, Green = Trial 2, Blue = Trial 3. (TIF) [file pone.0175430.s005.tif]

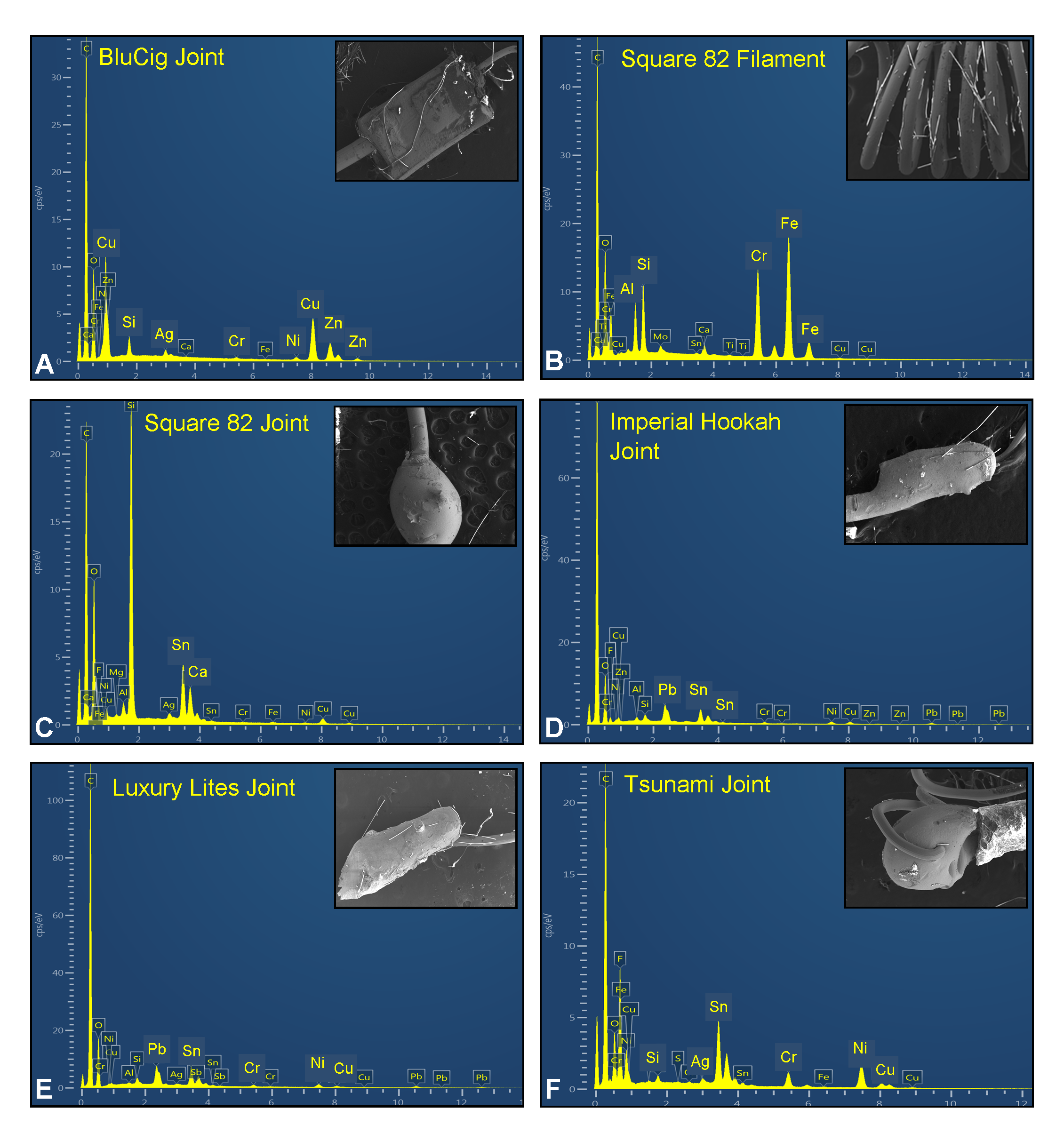

Supplement: S4 Fig — (A) BluCig thick wire to filament joint, (B) Square 82 filament, (C) Square 82 solder joint, (D) Imperial Hookah solder joint, (E) Luxury Lites solder joint, and (F) Tsunami solder joint. (TIF) [file pone.0175430.s006.tif]
